# Water-Resistant Antibacterial Coatings Using Cetylpyridinium Chloride - Graphene Oxide Composites

**DOI:** 10.1021/acsomega.5c10140

**Published:** 2026-02-26

**Authors:** Keisuke Okubo, Gen Kano, Masato Komoda, Kazuhiro Omori, Yuta Nishina, Shogo Takashiba

**Affiliations:** † Department of Periodontics and Endodontics, Field of Medical Development, 12997 Okayama University, 2-5-1 Shikata-cho, Kita-ku, Okayama 700-8525, Japan; ‡ Graduate School of Medicine, Dentistry and Pharmaceutical Sciences, Okayama University, 2-5-1 Shikata-cho, Kita-ku, Okayama 700-8525, Japan; § Research Institute for Interdisciplinary Science, Okayama University, 3-1-1 Tsushima-naka, Kita-ku, Okayama 700-8530, Japan; ∥ Department of Pathophysiology - Periodontal Science, Faculty of Medicine, Dentistry and Pharmaceutical Sciences, Okayama University, 2-5-1 Shikata-cho, Kita-ku, Okayama 700-8525, Japan

## Abstract

Hospital-acquired infections remain a persistent threat in healthcare settings, especially with the increasing number of elderly and immunocompromised patients. In situations where the use of disposable materials is difficult, durable antibacterial surface coatings are essential. In this study, we report the structural characterization of cetylpyridinium chloride-graphene oxide (CPC–GO) hybrid materials and the sustainability of their antibacterial effects, aiming at washable antibacterial coatings for medical applications. Graphene oxide (GO) has a large surface area and numerous functional groups, while cetylpyridinium chloride (CPC) is a quaternary ammonium compound with well-documented antibacterial activity. We hypothesized that the stable incorporation of CPC through the functional groups of GO could improve surface retention and provide long-term antibacterial performance. The structural properties of the CPC–GO composites were characterized by UV–vis spectroscopy, X-ray diffraction, thermogravimetric analysis, scanning electron microscopy, and atomic force microscopy. These analyses confirmed the formation of a complex through ionic bonds and the maintenance of a planar composite structure. The antibacterial performance of the CPC–GO coatings was examined using representative bacteria. Notably, the CPC–GO coatings maintained their antibacterial activity significantly better than the negative controls even after multiple washings. The excellent surface retention of the CPC–GO composite suggests its potential as a next-generation antibacterial coating for areas where disinfection and sterilization are impossible, such as the interior of complex medical devices. This study suggests a strategy to extend the efficacy of existing antibacterial agents through the application of nanomaterials. Future studies will focus on the controlled release, long-term stability, and biocompatibility of CPC to realize clinical applications.

## Graphical abstract

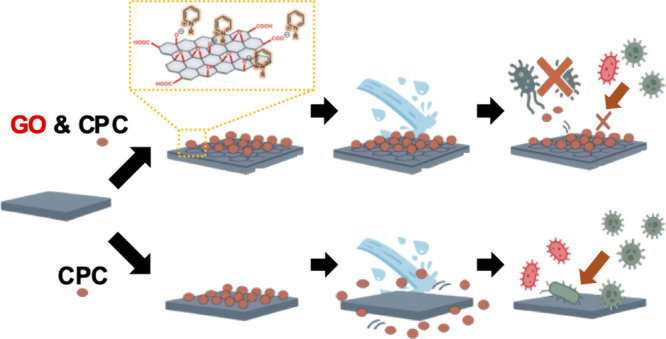

## Introduction

The response to nosocomial infections in medical institutions has become increasingly sophisticated, driven by shifts in the social and healthcare landscape, such as the aging population, the growing number of patients at high risk for infections, and the rising awareness among healthcare users.^1^ Despite these advancements, however, there remain critical challenges in clinical environments and with medical devices, where standard disinfection and sterilization procedures may not be feasible, or where disposable devices cannot be used for each patient.^2^ These gaps have the possibility to create some potential blind spots, leaving medical environments vulnerable to outbreaks of hospital-acquired infections. Developing materials with durable antimicrobial activity and broad applicability is essential to address these gaps. Effective prevention requires inhibiting microbial adhesion at the interface and sustaining antimicrobial function on frequently contacted surfaces.

Cetylpyridinium chloride (CPC), a quaternary ammonium compound, exhibits broad-spectrum antimicrobial activity by disrupting microbial lipid bilayers, which leads to cytoplasmic leakage and cell death.^3,4^ However, its practical application is hindered by weak adsorption to surfaces, resulting in a rapid loss of activity and limited durability. This underscores the need for improved strategies to enhance CPC retention on material surfaces.

Graphene oxide (GO) is a two-dimensional carbon material with abundant oxygen-containing functional groups and a high specific surface area (>2000 m^2^/g), enabling effective film formation and chemical functionalization.^5–7^ Furthermore, GO has been reported to exhibit strong interactions with organic and biomolecules. In particular, the complexation of GO with cationic polymers has been shown to enhance virus adsorption efficiency.^8^ The introduction of alkyl chains or amine functional groups onto GO further enables the effective capture and release of viruses such as Qβ phage, highlighting its potential as a virus-capturing material.^9^ In addition, GO can serve as a drug delivery carrier when combined with anticancer agents. Composite systems incorporating GO with biomolecules such as peptides and siRNA have enabled the construction of pH-responsive drug delivery systems,^10,11^ expanding its applicability to targeted molecular transport in biological environments. Moreover, recent review articles have demonstrated that practical large-scale production techniques for GO, including oxidation-based and electrochemical exfoliation methods, have already been well established.^12^ Therefore, GO’s biocompatibility and scalable synthesis further support its biomedical potential.^6,13,14^

Previous work demonstrated that CPC exhibits sustained release from GO, modulated by CPC’s terminal functional groups.^15^ However, comprehensive structural characterization of CPC–GO composites and their wash-resistant antibacterial efficacy remain underexplored. Recently, Miyaji et al. reported multilayered GO–CPC coatings that exhibited sustained antibacterial effects in wet environments.^16^ While, their coating system required layer-by-layer deposition and polymer-assisted assembly. In contrast, the present study focuses on a simpler and binder-free synthesis strategy, where CPC and GO form hybrid complexes in a one-step aqueous process through direct electrostatic interaction. This approach enables uniform hybridization without multilayer processing and can be readily applied to various substrates. Furthermore, we systematically elucidated the intermolecular interaction between CPC and GO and its influence on CPC retention and wash resistance, which were not mechanistically clarified in previous reports.

In this study, we systematically characterize the structure of CPC–GO composites by employing ultraviolet–visible spectroscopy (UV–Vis), Fourier-transform infrared spectroscopy (FT–IR), X-ray diffraction (XRD), thermogravimetric analysis (TGA), scanning electron microscopy (SEM), and atomic force microscopy (AFM) to elucidate their composition, bonding interactions, and morphology. We also evaluate their antibacterial efficacy through a series of in vitro assays, including bacterial viability tests and adenosine triphosphate (ATP) quantification, to assess both initial activity and retention of antibacterial function after repeated washing. Our results reveal the underlying molecular interactions that facilitate CPC retention on GO surfaces and demonstrate the potential of CPC–GO composites as durable, wash-resistant antibacterial coatings for reusable medical devices. This approach offers a promising strategy for reducing the risk of nosocomial infections in clinical settings.

## Results and Discussion

### Structural Characterization of CPC–GO Composites: Quantification, Thermal Stability, and Interlayer Expansion

To prepare the CPC–GO composite, the optimal weight ratio of CPC to GO was first determined. First, the absorbance of each CPC solution prepared at concentrations ranging from 3 × 10^–3^ to 5 × 10^–2^ mg/mL was measured using UV–vis (Figure 1a). A calibration curve was then created based on the absorbance of each concentration at 258 nm, corresponding to the absorbance of the pyridine ring that CPC possesses (Figure 1b). To estimate the amount of CPC incorporated into the CPC–GO composite, GO was added to a CPC aqueous solution at a CPC–GO weight ratio of 1:1, resulting in the adsorption of CPC onto the GO surface and a corresponding decrease in the CPC concentration in the solution. The mixture was then centrifuged to separate the CPC–GO composite, and the residual CPC remaining in the supernatant was quantified using the calibration curve, as shown in red dot. As a result, more than 95% of CPC was introduced when the weight ratio of CPC–GO ratio was 1:1. CPC–GO prepared based on this weight ratio was used in the following experiment.

**Figure 1:**
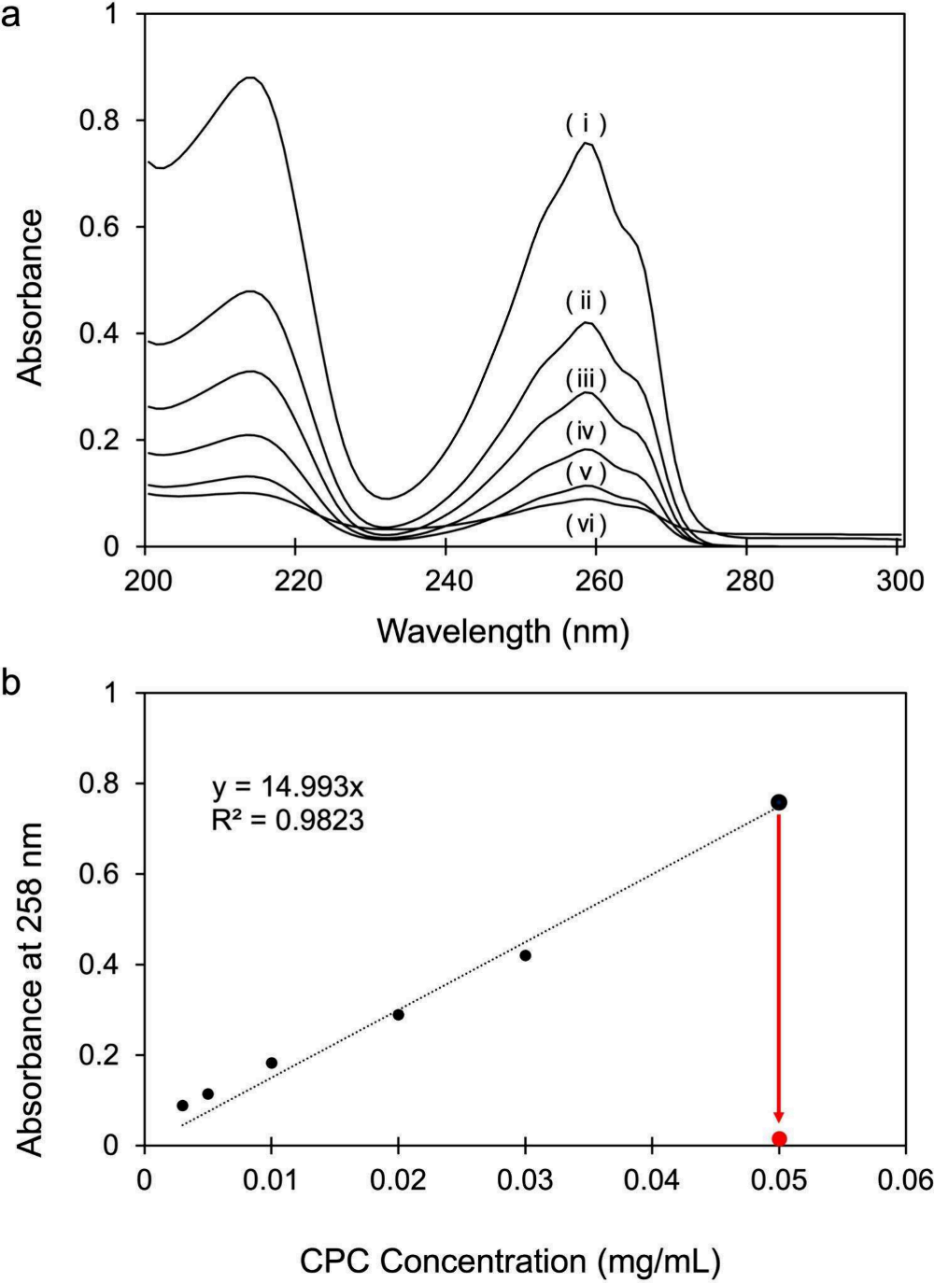
UV–vis analysis of [a] CPC with different concentrations, and [b] calibration curve of CPC (black) and supernatant solution of CPC–GO = 1:1 (red). CPC concentration is (i) 0.05, (ii) 0.03, (iii) 0.02, (iv) 0.01, (v) 0.005, (vi) 0.003 mg/mL.

The incorporation of CPC into the GO structure was further analyzed by thermogravimetric analysis (TGA), as shown in Figure 2. The TGA curve of pure CPC exhibited a major decomposition event around 300 °C (Figure 2; Region A in (iii)), corresponding to the thermal degradation. In contrast, GO alone showed a gradual weight loss of 35% up to 200 °C (Figure 2; Region B in (ii)), which is attributable to the thermal removal of oxygen-containing functional groups.^17^ The CPC–GO composite displayed two distinct weight loss regions: one around 200 °C (25% loss), similar to GO, and a second between 300 and 500 °C (19% loss) (Figure 2; Area C in (i)). The second region of weight loss was not observed in GO alone and is attributable to the decomposition of CPC incorporated into the composite. However, despite the presence of CPC in the composite at an approximate 1:1 weight ratio to GO, the total weight loss of CPC–GO (Figure 2; Region D in (i)) was nearly identical to that of GO alone, and significantly less than the 100% decomposition observed for pure CPC. Moreover, the decomposition profile of CPC in the composite differs markedly from that of CPC alone. These findings suggest that CPC is thermally stabilized when incorporated into GO, likely due to strong interactions between CPC and the GO surface. Such interactions may involve physical confinement between GO layers or electrostatic and π–π interactions that restrict molecular motion and inhibit volatilization during heating. This enhanced thermal stability supports the successful integration of CPC into the GO matrix, beyond simple physical mixing.

**Figure 2:**
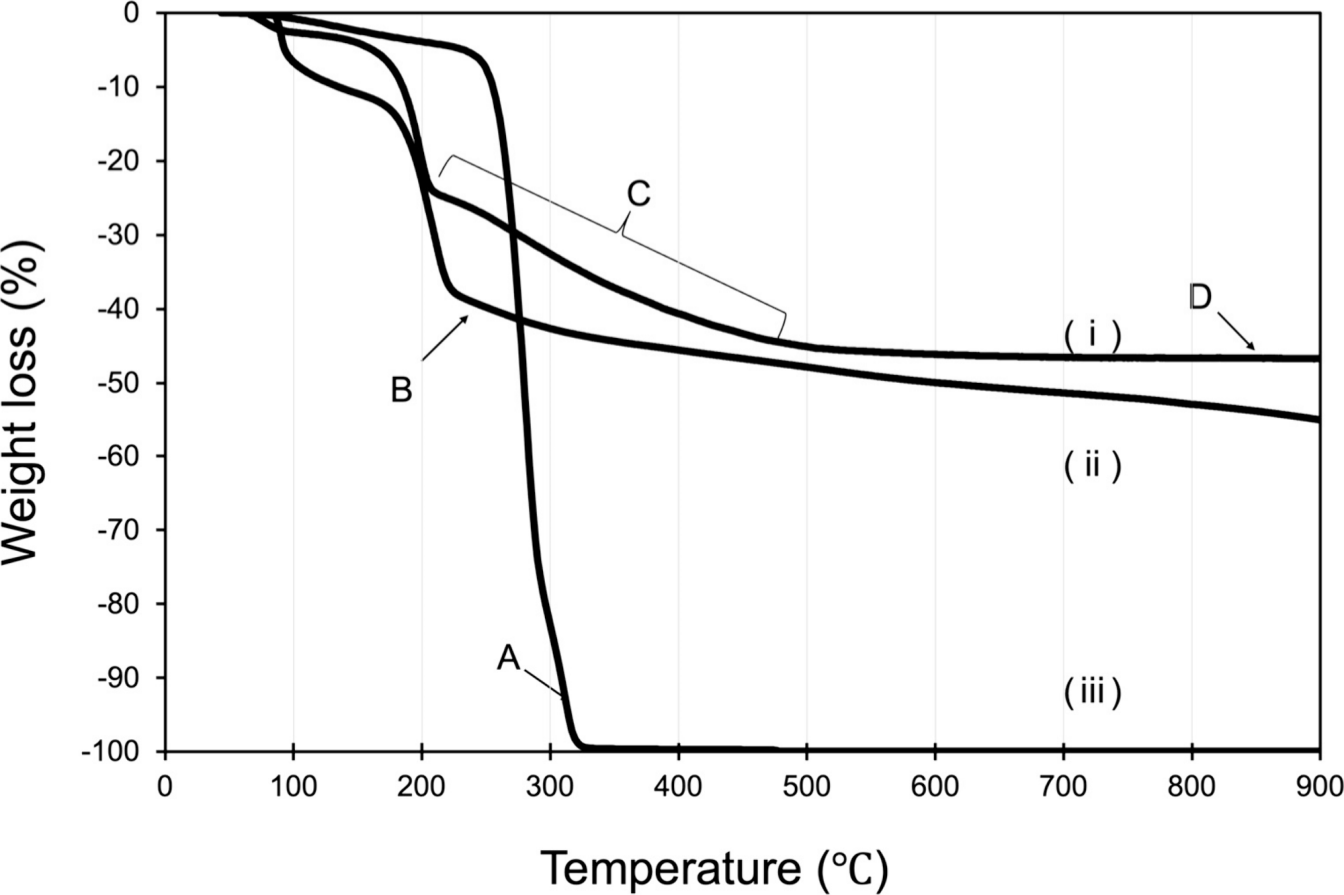
TGA analysis of (i) CPC–GO, (ii) GO, and (iii) CPC.

The lattice structure of CPC–GO was analyzed by XRD, as shown in Figure 3. The interlayer spacing (d) of each material was calculated using Bragg’s equation, 2d sin θ = λ, where θ is the diffraction angle and λ is the wavelength of the incident X-ray (Cu Kα, λ = 1.5418 Å). The diffraction patterns in the range of 2θ = 8.0 – 12.0° (Figure 3; Area A in (i)) correspond to the periodicity between GO layers and shift to lower angles with increasing interlayer distance. The XRD pattern of CPC–GO showed a distinct peak at 2θ = 8.02° (Figure 3; Region B in (ii)), which was different from those of both GO and pure CPC. This observation indicates that CPC–GO does not contain crystalline CPC domains and suggests that CPC is incorporated into GO layers. Compared to the diffraction peak of GO (2θ = 11.2°, corresponding to an interlayer distance of 7.89 Å), the CPC–GO peak position indicates an expanded interlayer spacing of 11.02 Å. This increase of 3.13 Å implies that CPC molecules are intercalated between GO layers. The observed shift to lower diffraction angles further supports the conclusion that CPC has been successfully introduced into the GO framework, resulting in a structural expansion consistent with interlayer incorporation.

**Figure 3:**
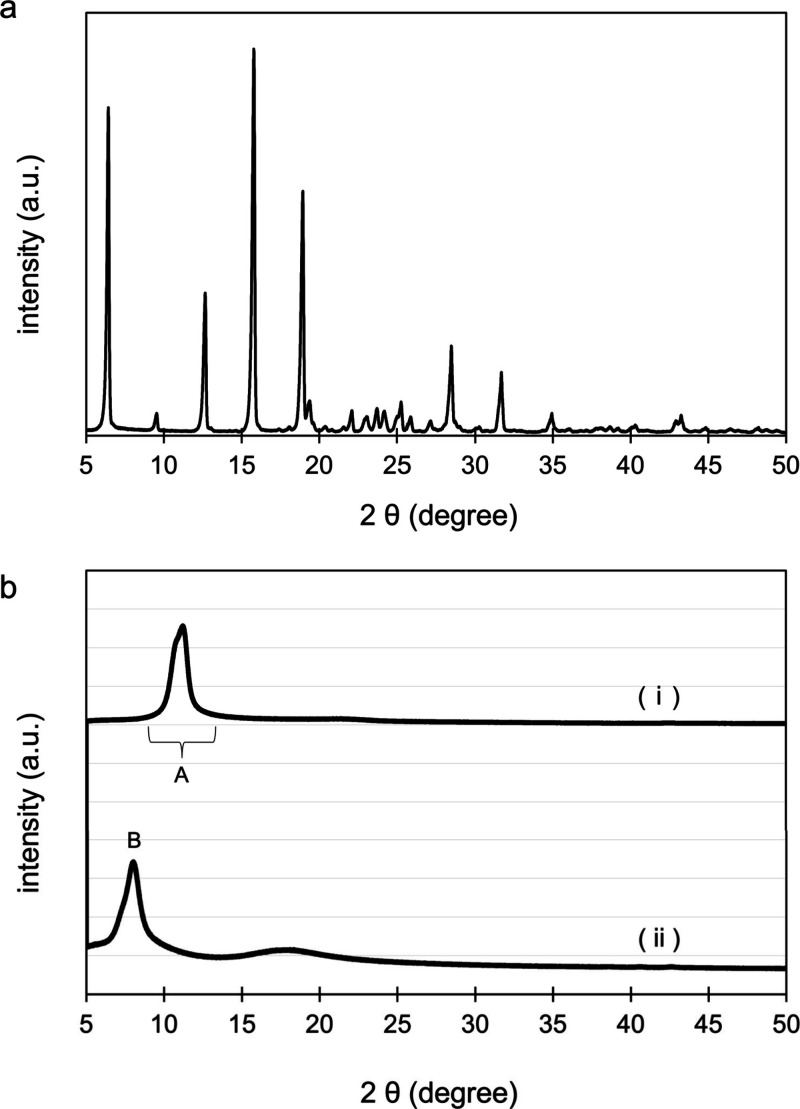
XRD analysis of [a] CPC, and [b] (i) GO and (ii) CPC–GO.

### Morphological Observation of CPC–GO and Bonding Mode between CPC and GO

The morphology of CPC–GO composites was investigated to assess changes in lateral dimensions, layer stacking, and thickness resulting from CPC incorporation. SEM analysis (Figure 4ab; (i)) revealed that both pristine GO and CPC–GO samples consisted of nanosheets with lateral sizes on the micrometer scale. In both cases, a mixture of monolayer and few-layer sheet structures was observed, with no significant difference in planar dimensions between the two materials. To evaluate thickness variations resulting from CPC adsorption, AFM was conducted on isolated nanosheets deposited on silicon substrates (Figure 4a,b; (ii), (iii)). While pristine GO exhibited a typical monolayer thickness of approximately 0.88 nm, consistent with literature values for exfoliated GO sheets,^18^ the CPC–GO samples showed an average thickness of 1.08 nm (Figure 4b; (iii)). This increase of approximately 0.2 nm suggests the successful adsorption of CPC molecules onto the GO surface, likely through electrostatic interactions and/or hydrophobic association involving CPC’s alkyl chains. Importantly, the observed increase in height was uniform across the monolayer regions of CPC–GO, indicating that the CPC molecules formed a relatively consistent layer rather than aggregating as discrete domains, as supported by XRD analysis. This morphological evidence further supports the conclusion that CPC was uniformly incorporated onto the GO nanosheets, contributing to their structural expansion as previously observed in the XRD analysis (Figure 3).

**Figure 4:**
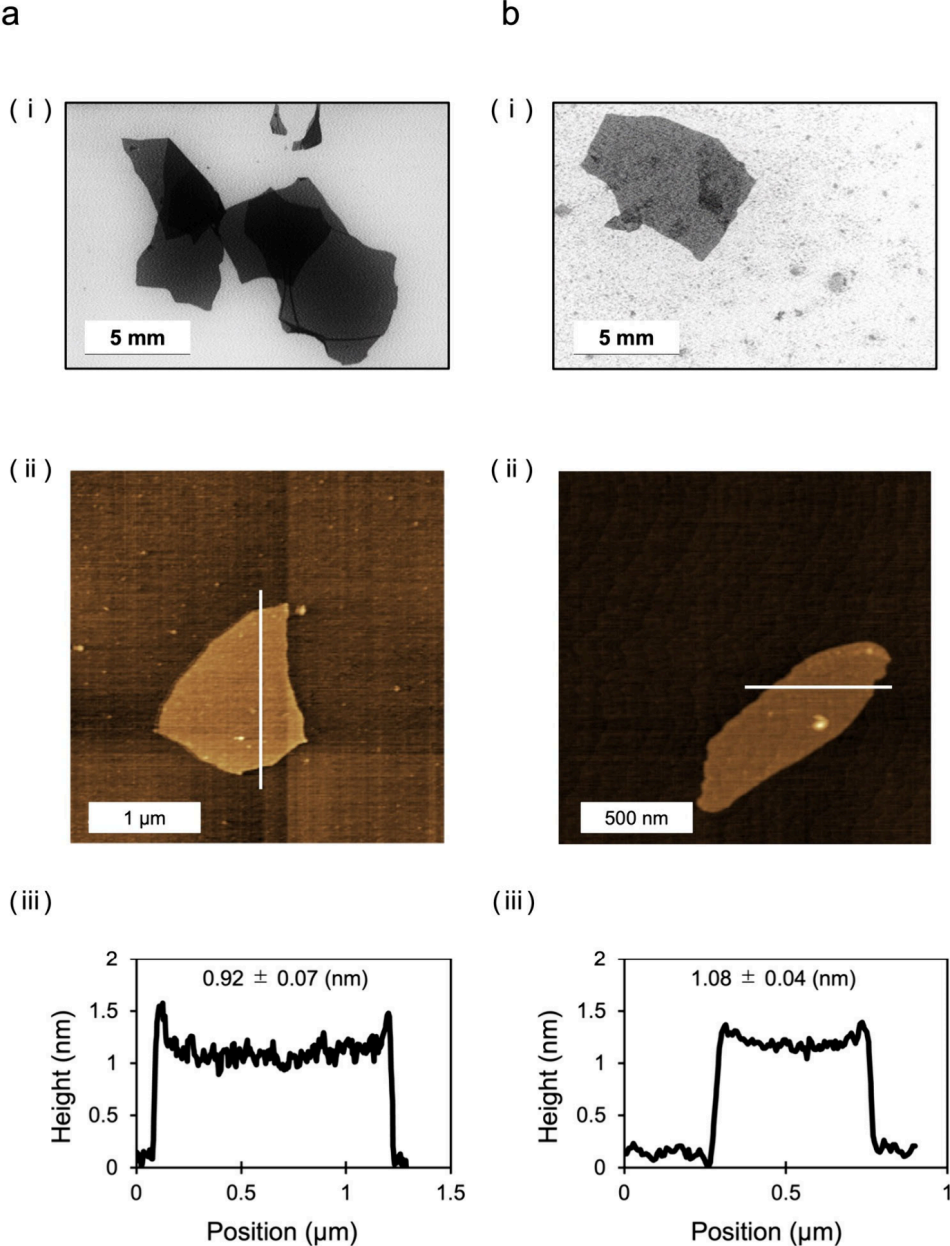
Morphological observations of [a] GO and [b] CPC–GO. (i) SEM, (ii) AFM, and (iii) AFM cross-section analysis.

To elucidate the nature of the interaction between CPC and GO, FT-IR measurement was conducted. FT–IR spectra of GO, CPC, and CPC–GO are shown in Figure 5. The CPC–GO spectrum exhibited a broad absorption band between 2900 and 3700 cm^–1^ (Figure 5; Area A in (i) and (iii)) corresponding to O–H stretching vibrations from hydroxyl groups in GO, and a distinct peak at approximately 2850 cm^–1^ (Figure 5; Region B in (i) and (ii)) attributable to the C–H stretching vibrations of the alkyl chain in CPC. These spectral features confirm the successful incorporation of CPC into the GO matrix. Notably, the characteristic peaks of oxygen functional groups on GO, such as the hydroxyl (2900–3700 cm^–1^: Figure 5; Area A in (i) and (iii)) and carbonyl (C=O, ∼1750 cm^–1^: Figure 5; Region C in (i) and (ii)) groups, did not exhibit significant shifts upon CPC loading. This suggests that the interaction between CPC and GO occurs primarily through noncovalent interactions rather than covalent bonding. Such interactions may enable the sustained release of CPC, a feature that is desirable for long-term antimicrobial functionality.

**Figure 5:**
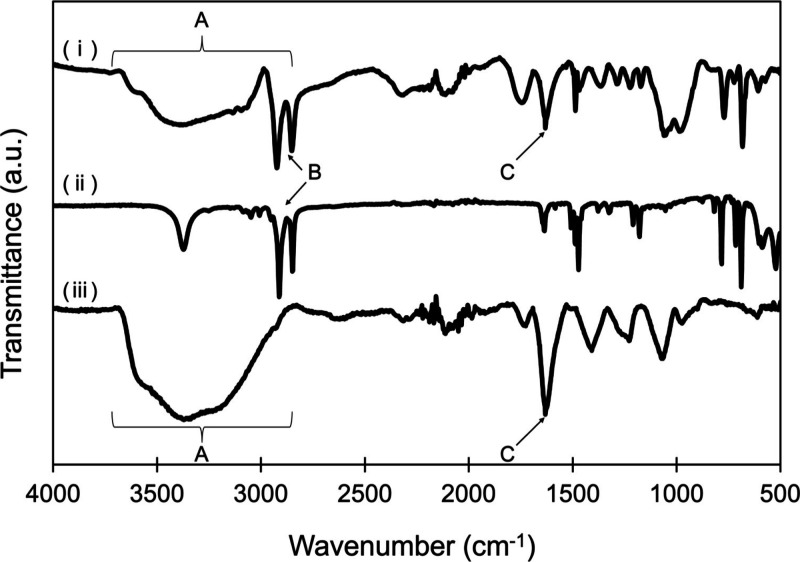
FT-IR spectra of (i) CPC–GO, (ii) CPC, and (iii) GO. (A) Region A (2900–3700 cm^–1^) corresponds to O–H stretching vibrations, (B) region B (∼2850 cm^–1^) to C–H stretching vibrations, and (C) region C (∼1750 cm^–1^) to C=O stretching vibrations.

To complement the FT–IR data and clarify the surface bonding environment, XPS measurements were performed on CPC, GO, and CPC–GO samples (Figure 6, Table 1). In pure CPC, the nitrogen-to-chlorine (N:Cl) atomic ratio was approximately 1:1, as expected from its chemical composition. However, after hybridization with GO, the Cl signal significantly decreased while the N 1s signal remained prominent. This change suggests the formation of ionic bonds between the positively charged pyridinium nitrogen of CPC and the negatively charged functional groups of GO, with concurrent release of chloride ions into solution. The decrease in Cl content in CPC after hybridization with GO further supports that the interaction is primarily ionic in nature.^19,20^ Zeta potential analysis can provide additional evidence for such interactions; however, in our case, aggregation occurred upon mixing CPC and GO, making the zeta potential results unreliable. The N 1s core-level spectrum of CPC–GO showed a peak consistent with quaternary ammonium nitrogen (+NC4), indicating that CPC retained its chemical identity after incorporation. Notably, the O 1s spectrum of CPC–GO also showed subtle changes compared to that of pristine GO. While the overall binding energy position remained largely unchanged, a slight decrease in the relative intensity of the high-binding-energy component (typically assigned to carboxyl and carbonyl oxygen species (Figure 6; (iii)) was observed. This may reflect a change in the local electronic environment of oxygen atoms upon electrostatic complexation with CPC. Taken together, these XPS results strongly support the conclusion that CPC is electrostatically adsorbed onto GO surfaces via interactions between pyridinium cations and negatively charged oxygen-containing groups, without inducing major covalent modification of the GO framework. It should be noted that the CPC content estimated from the N 1s signal intensity in XPS was higher than the CPC retention value calculated from the UV–Vis measurement after washing. This discrepancy arises from the differences in what each technique measures. XPS detects all nitrogen species present on the surface, including both strongly bound CPC and weakly adsorbed or physically associated CPC that can be removed during the washing process. In contrast, UV–Vis quantifies only the amount of CPC that remained on GO after washing, corresponding to the strongly bound fraction. Therefore, the difference between the two values reflects the coexistence of multiple binding states of CPC on GO, rather than incomplete loading. This interpretation is further supported by the TGA results (Figure 2), in which CPC in the CPC–GO composite exhibited a higher thermal stability than free CPC, indicating strong interaction with the GO framework.

**Table 1:** Elemental Analysis of GO, CPC, and CPC–GO by XPS

	C (at %)	O (at %)	N (at %)	Cl (at %)
GO	69.2	30.9		
CPC	84.2	10.1	2.8	2.8
CPC–GO	72.5	25.8	1.4	0.2

**Figure 6:**
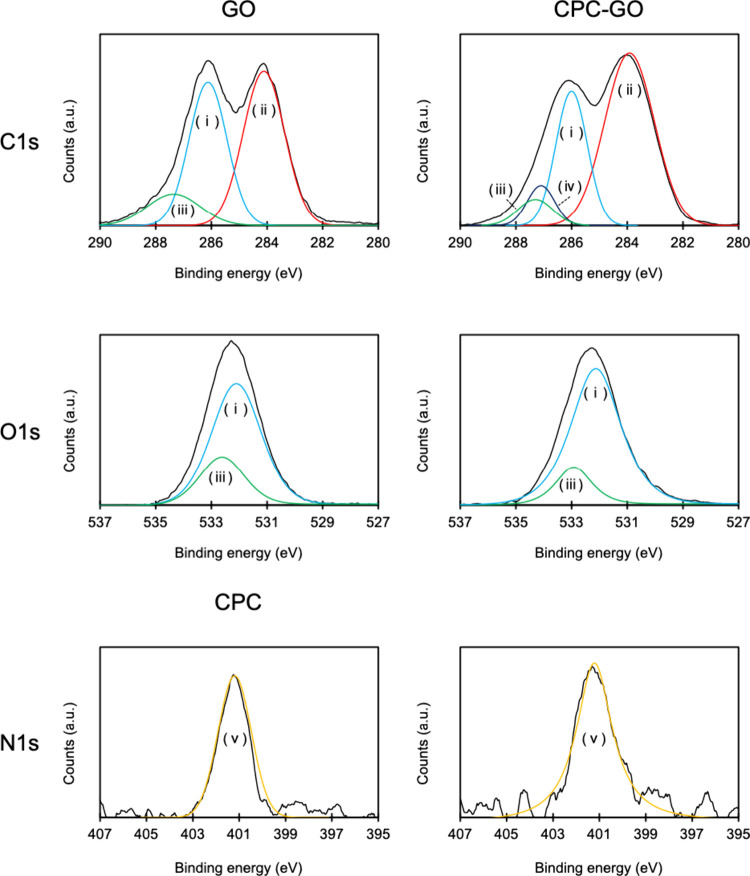
XPS analysis of GO and CPC–GO at C 1s, O 1s, and of CPC at N 1s regions. Line colors: (i) C–O, Blue; (ii) C–C, Red; (iii) C=O, Green; (iv) C–N, purple; (v) +NC4, Yellow.

### Investigation of Effective Concentration of CPC–GO

The CPC concentration at which CPC–GO could exert its antimicrobial effect was examined (Figure 7). *Streptococcus mutans* (*S. mutans*), which served as a representative bacterium in this experiment, is one of the oral bacteria, which is tolerant even in acidic conditions.^21^ The results showed that CPC–GO at concentrations of 0.05% (w/v) or higher significantly inhibited bacterial growth even after two times washings compared to the negative control group. In other words, after two consecutive washing, even CPC at twice the maximum concentration of 0.1% (w/v) which is currently recommended by the Scientific Committee on Consumer Safety^22^ fails to retain antibacterial activity, whereas CPC–GO containing CPC at concentrations of 0.05% (w/v) or higher maintains its antibacterial efficacy significantly (Figure 7a). Moreover, the color of the bacterial solution after incubation was noticeably clear at the wells that treated by CPC–GO containing CPC at concentrations of 0.05% (w/v) or higher. (Figure 7b) These results suggest that the synergistic effect of the surface retention properties of CPC–GO as a nanomaterial and the strong antibacterial activity of CPC.

**Figure 7:**
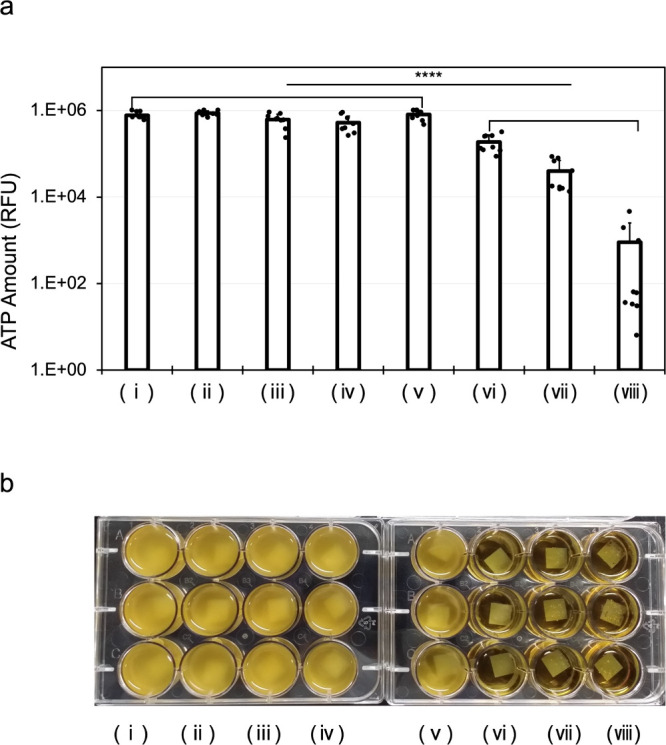
Antibacterial effect against *S. mutans*. These samples were as follows: (i) PBS, (ii) 0.2% GO, (iii) 0.01% CPC, (iv) 0.2% CPC, (v) 0.01% CPC, (vi) 0.05% CPC–GO, (vii) 0.1% CPC–GO, (viii) 0.2% CPC–GO. [a] Bacterial ATP assay data. Data are presented as mean ± standard deviation (*N* = 9). Statistical analysis was conducted using one-way ANOVA; **** indicates *P* < 0.0001. [b] Representative photograph taken after 12 h of incubation. These experiments were independently repeated three times.

In this study, *S. mutans* was chosen as the test organism because it is a representative Gram-positive bacterium possessing a thick peptidoglycan cell wall, which provides structural robustness and resistance to external stress. However, other clinically important Gram-positive pathogens involved in hospital-acquired infections, such as methicillin-resistant *Staphylococcus aureus* or *Enterococcus* spp., were not included in this study. This represents a limitation, and future investigations should evaluate whether CPC–GO can exert similar sustained antimicrobial effects against these opportunistic pathogens, thereby broadening its clinical relevance. Furthermore, in this study, silicone substrates were used as a model surface to enable reproducible coating formation and antibacterial evaluation under controlled conditions. We acknowledge that the surface energy and chemical properties of different clinically used substrate materials, such as metals and polymers, may influence coating adhesion and performance. A systematic evaluation of various medical device materials will be conducted in future work.

## Conclusions

CPC was bound to GO at a ratio of approximately 1:1 by weight, and the mode of binding was ionic bonding. And more, CPC–GO could maintain its antimicrobial effect on the surface of a silicon sheet even after two times washings. In the future, it is necessary to verify the antimicrobial effect using test specimens other than silicon, to analyze the adhesion mechanism at the interface between CPC–GO and the test specimens, and to clarify the mechanism by which CPC–GO exerts sustained antimicrobial activity.

## Experimental Materials and Methods

### Preparation of GO and CPC–GO

An aqueous dispersion of GO nanosheets was prepared according to a previously reported method developed by our group.^23^ The CPC–GO composite was obtained by reacting GO with CPC (Sigma-Aldrich, St. Louis, MO, USA). Specifically, 20 mL of an 8.4 mg/mL GO aqueous dispersion was mixed with CPC at a final concentration of 1.0 mg/mL under vigorous stirring, followed by mild sonication using a bath-type sonicator for 30 min. The resulting mixture was then freeze-dried (DRZ350WC: ADVANTEC, Tokyo, Japan), as described previously.^15^ All solutions were prepared using ultrapure water obtained from a Milli-Q IQ 7003 purification system (Millipore).

### Spectroscopic Analysis

The incorporation rate of CPC in the GO composite was estimated using a UV–vis-NIR spectrophotometer (V-670; JASCO, Tokyo, Japan). The chemical bonding in the GO composite was analyzed by Fourier transform infrared spectroscopy (FT-IR) with an attenuated total reflectance (ATR) unit (IR Tracer 100; Shimadzu Corporation, Tokyo, Japan) using powder samples prepared by freeze-drying the GO composite.

### Structural and Surface Characterization

The lattice structure of the GO composite was analyzed by powder X-ray diffraction (XRD) using a PANalytical X’Pert PRO diffractometer with Cu Kα radiation (λ = 1.541 Å) over a 2θ range of 5–75°. The operating current and voltage were 30 mA and 40 kV, respectively. Data were collected continuously with a step size of 0.017°. The morphology of the GO composite was observed using a scanning electron microscope (SEM; Hitachi S-5200) operated at an acceleration voltage of 20 kV. Surface thickness measurements were performed with an atomic force microscope (AFM; Shimadzu SPM-9700HT). Elemental surface composition (C, O, N, Cl) was determined by X-ray photoelectron spectroscopy (XPS; JPS-9030, JASCO) with a pass energy of 20 eV.

### Thermal Analysis

The incorporation ratio of CPC in the GO composite was estimated based on the weight loss profile obtained by thermogravimetric analysis (TGA) of freeze-dried CPC–GO powders. TGA measurements were performed using a Thermo plus EVO2 instrument (Rigaku, Tokyo, Japan).

### Test Specimens and Treatments for These Surfaces

Silicon sheets (10 × 10 × 1 mm; Sansho, Tokyo, Japan) were used as substrates for coating with GO test solutions. Prior to coating, the silicon sheets were silanized by immersion in 0.5 mL of 10% (w/v) 3-(trimethoxysilyl)­propyl methacrylate (Tokyo Kasei Kogyo, Tokyo, Japan) for 2 h. After removing excess silane solution by air drying, the silanized silicon sheets were immersed in 0.5 mL of the GO test solution for 30 min. The coated specimens were then washed twice with 4 mL of ultrapure water.

### Bacteria

*S. mutans* ATCC 25175 strain (ATCC, USA) was cultured aerobically in brain heart infusion (BHI) broth (Becton, Dickinson and Company, Sparks, MD, USA) at 37 °C for 6 h to reach the logarithmic growth phase. The bacterial suspension was then diluted with fresh BHI medium to a concentration of 1.0 × 10^5^ CFU/mL. Bacterial turbidity in each test solution was measured using a photometer (Miniphoto 518R; Taitec, Saitama, Japan) at a wavelength of 660 nm.^15^ And then, 4 mL of the bacterial solution was used per specimen and incubated at 37 °C for 12 h.

### Measurement of the Amount of Adenosine Triphosphate (ATP)

Bacterial viability was evaluated by measuring intracellular ATP levels. After 12 h of exposure to the test solutions, ATP content was determined using the Lucifer HS kit in combination with a Lumi-tester C-110 (Kikkoman Bio-Chemiphar, Tokyo, Japan), based on the luciferin–luciferase bioluminescence reaction. The luminescence output was expressed in relative light units (RLU), corresponding to a detection range of 1.0 × 10^–16^ to 3.0 × 10^–11^ mol of ATP. To remove extracellular ATP prior to measurement, adenosine phosphate deaminase was added.^15,24,25^

### Statistical Analysis

Statistical analysis was performed using one-way analysis of variance (ANOVA), followed by Tukey’s multiple comparisons test. All analyses were conducted using GraphPad Prism, version 8.4.3 (686) (GraphPad Software, San Diego, CA). Statistical significance was defined as *p* < 0.05.

## Abbreviations

GO: graphene oxide
CPC: cetylpyridinium chloride.
